# Supplementary material for: Piezo1 Is Required for Myoblast Migration and Involves Polarized Clustering in Association with Cholesterol and GM1 Ganglioside
Source: Cells. 2023 Dec 7;12(24):2784. doi: 10.3390/cells12242784 (PMC10741634; doi:10.3390/cells12242784)
Supplement: Supplementary file 1 [file cells-12-02784-s001.zip › cells-2639434-supplementary.pdf]

cells

MDPI

Supplementary Materials

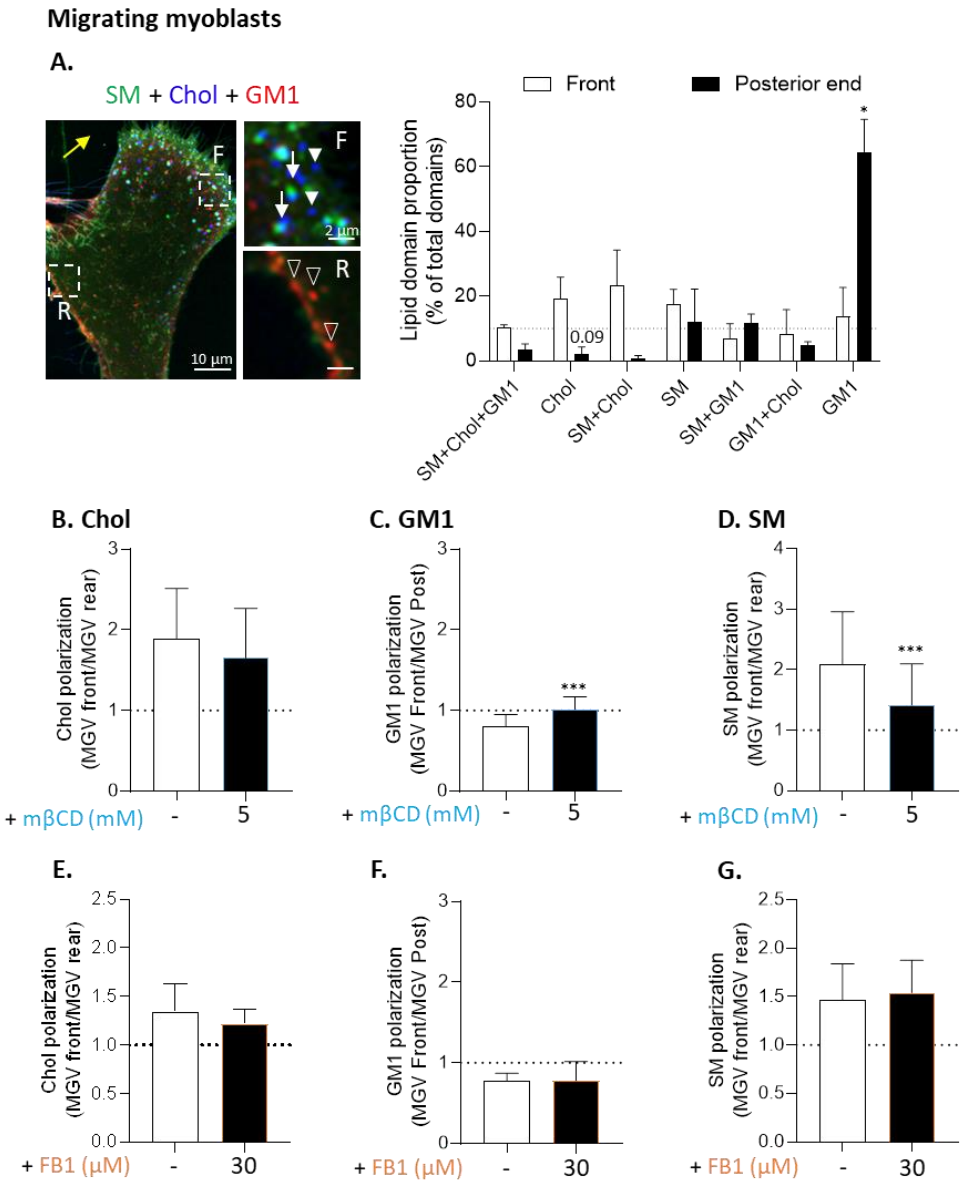

Figure S1. Membrane cholesterol and sphingomyelin polarize at the front and GM1 ganglioside at the rear upon myoblast migration and cluster in different types of lipid domains. (**A**) Image of a migrating cell triple-labeled for SM, chol and GM1 and quantification of lipid domain proportion at the front vs. the rear. Adapted from [27]. F, migration front; R, rear; yellow arrow, direction of migration. (**B-G**) Quantification of chol (B,E) and SM polarization (D,G) after treatment with mβCD or FB1 on migrating cells. Adapted from [27]. The same was done here for GM1 polarization: cells were single-labeled for GM1 (CTxB) after 5 hours of migration and pretreatment with 5 mM mβCD (C) or 30 µM FB1 (F). Data are expressed as the ratio of the MGV at the front *vs.* the MGV at the rear (*n*= 52-53 cells from 6 independent experiments for mβCD and 8 cells from 1 experiment for FB1). Unpaired t-test.

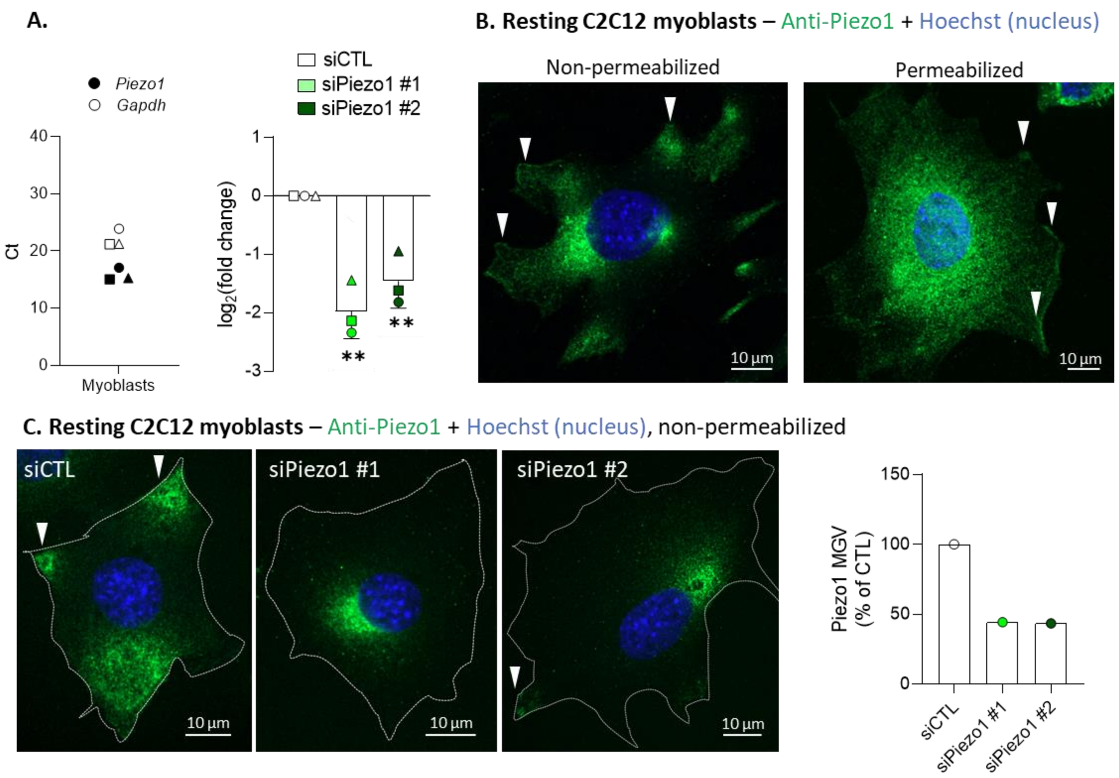

Figure S2. Piezo1 silencing by siRNA in C2C12 myoblasts diminishes the Piezo1 surface distribution. (**A**) Ct from a qPCR of *Piezo1* and *Gapdh* (housekeeping gene) on myoblasts (left graph, $n = 3$ independent experiments). Gene expression of *Piezo1* was evaluated after transfection with two different siRNAs targeting Piezo1 and compared to a negative control siRNA. Data are expressed as base-2 logarithm of fold change (right graph, $n = 3$ independent experiments). (**B**,**C**) Myoblasts were directly fixed (B) or were first transfected with a negative control siRNA or with two different siRNAs targeting Piezo1 (C) then immunolabeled for Piezo1 (green) and nucleus (blue) in non-permeabilized or in permeabilized conditions, as indicated above the panels, and visualized by confocal microscopy. Arrowheads indicate Piezo1 in membrane protrusions. Quantification of Piezo1 fluorescence MGV was performed to determine the efficiency of the siRNAs ($n = 1$ experiment). One-way ANOVA followed by Dunnett's multiple comparisons test.

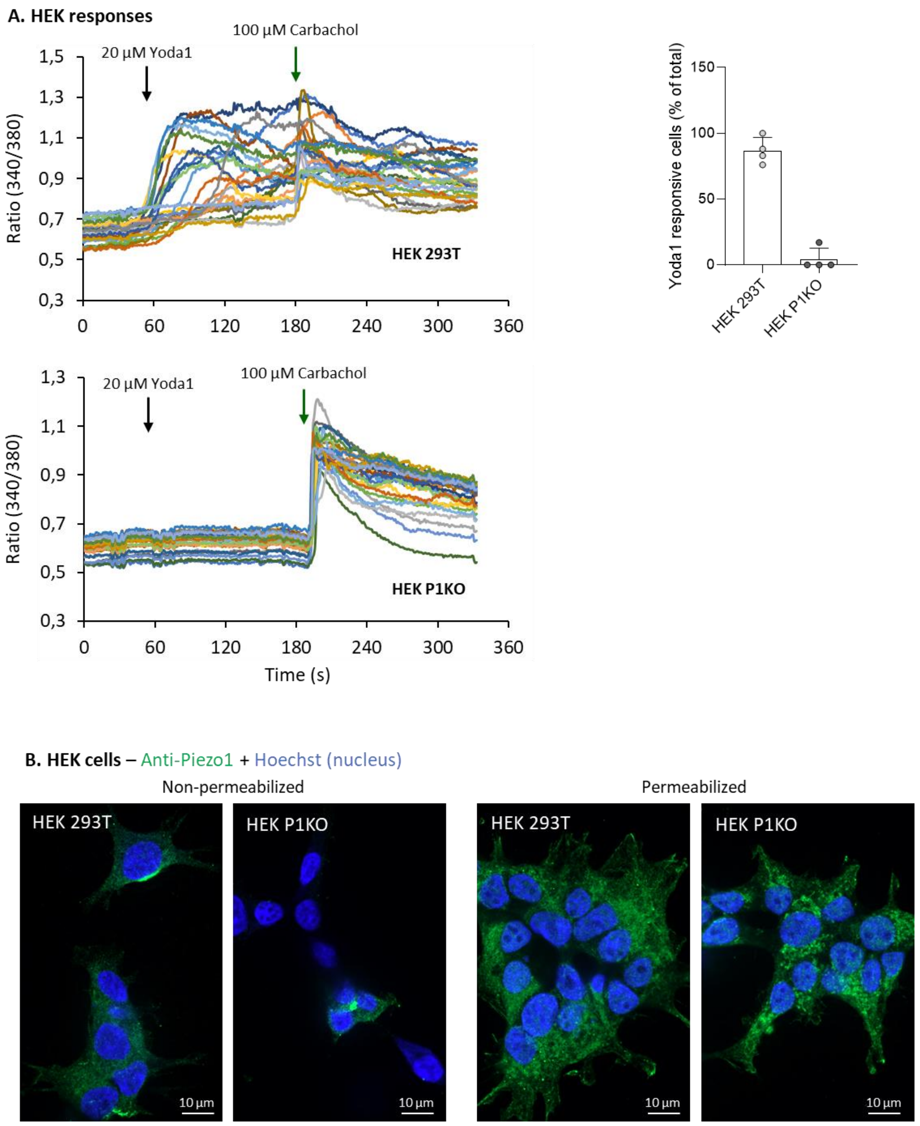

Figure S3. Knock-out HEK cells present a strong decrease of their responsiveness to activation by Yoda1 and of Piezo1 surface distribution. (**A**) Proportion of cells showing a Ca^{2+} response after 90 seconds of treatment with 20 µM Yoda1 followed by 100 µM Carbachol without washing used as positive control (n = 25-30 cells from 4 independent experiments). (**B**) Control and Piezo1 KO HEK cells were immunolabeled for Piezo1 (green) and nucleus (blue) in non-permeabilized or permeabilized conditions and visualized by confocal microscopy (n = 1 experiment).

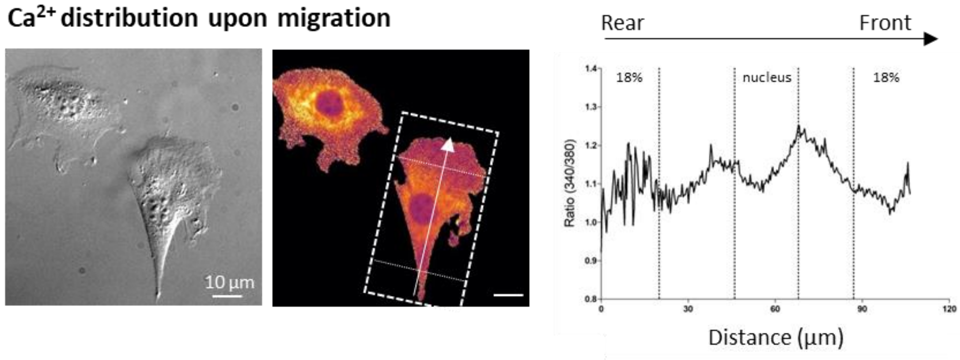

Figure S4. Ca^{2+} distribution is slightly higher in the center of myoblasts upon migration. Representative pseudocolors image of Fura2 ratio (340/380) and brightfield image of myoblasts with an

example of Fura2 ratio (340/380) intensity profile from rear to front of one individual migrating cell. Dash lines show the first and last 18% of cell total length used to measure the polarization of Fura2 signal.

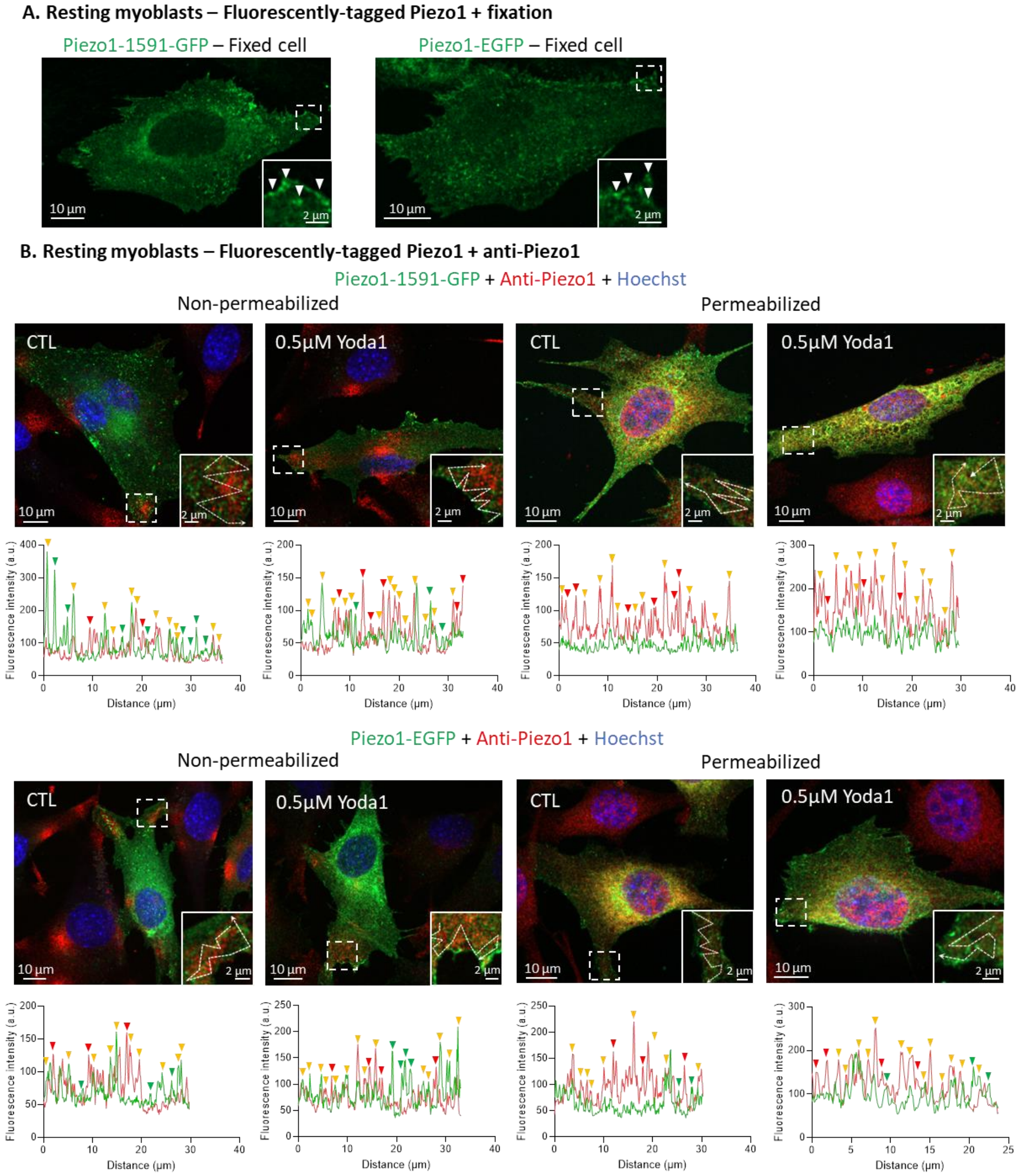

Figure S5. Fluorescently-tagged Piezo1 is not affected by cell fixation and partially colocalizes with endogenous Piezo1. (**A**,**B**) Cells were transfected with either Piezo1-1591-GFP or Piezo1-EGFP, left untreated or pretreated with 0.5 µM Yoda1 for 30 minutes, fixed, then immediately visualized by vital confocal microscopy (A) or (immuno)labeled for Piezo1 (red) and nucleus (blue) with or without cell permeabilization (B). Fluorescence intensity profiles were drawn to visualize the overlap between Piezo1 fluorescent construct and antibody. Arrowheads, Piezo1 clusters; dotted arrows in insets, profiles; green and red arrowheads, Piezo1 construct and Piezo1 antibody clusters; yellow arrowheads, colocalization.

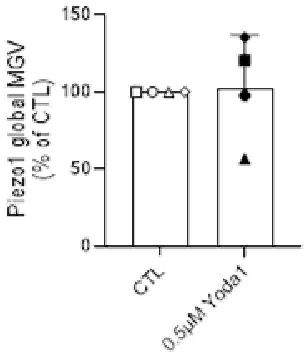

Figure S6. Piezo1 global fluorescence intensity in migrating myoblasts does not change upon its activation with Yoda1. Quantification of Piezo1 global fluorescence via the MGV of the entire CTL and Yoda1-treated myoblasts from images presented in Figure 3C. Data are expressed as % of control (*n* = 4 independent experiments). One sample t-test.

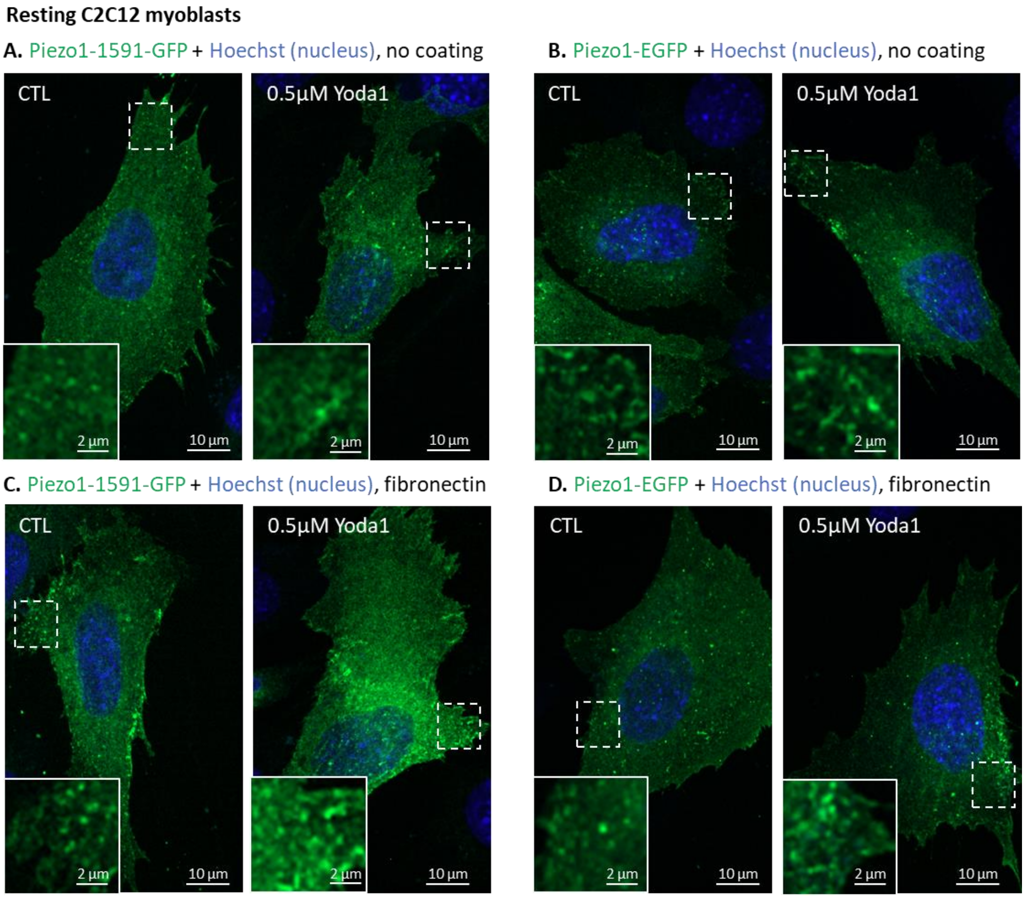

Figure S7. Surface Piezo1 clusters are observed on both myoblasts seeded on uncoated or fibronectin-coated coverslips. Cells were seeded on uncoated (A,B) or fibronectin-coated (C,D) coverslips then transfected with two different Piezo1 fluorescent constructs (Piezo1-1591-GFP in A,C and Piezo1-EGFP in B,D) and treated or not with 0.5 μM Yoda1 for 30 minutes. Cells were then fixed, labeled for the nucleus (blue) and visualized by confocal microscopy (representative images of 1 experiment). Insets show Piezo1 in membrane protrusions.

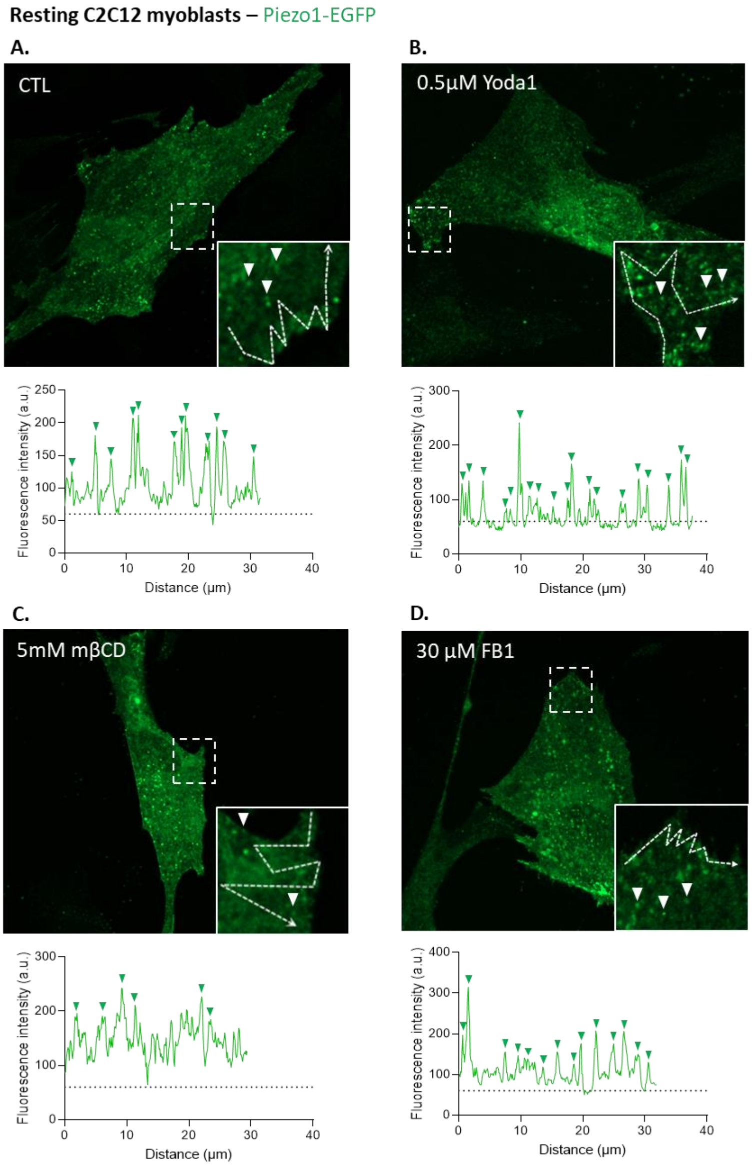

Figure S8. Cholesterol depletion, but not sphingolipid depletion, decreases the number of fluorescent peaks at the periphery of myoblasts expressing a fluorescent Piezo1 construct – Extension of Figure 4A. Myoblasts were transfected with the fluorescent Piezo1-EGFP construct (A) then treated for 30 minutes with 0.5 μM Yoda1 (B) or 5 mM mβCD (C) or for 48h with 30 μM FB1 (D) and visualized by vital confocal microscopy. Fluorescence intensity profiles were drawn along the cell periphery in protrusions to visualize Piezo1 clusters abundance. Arrowheads, Piezo1 clusters; dotted arrows, profile.

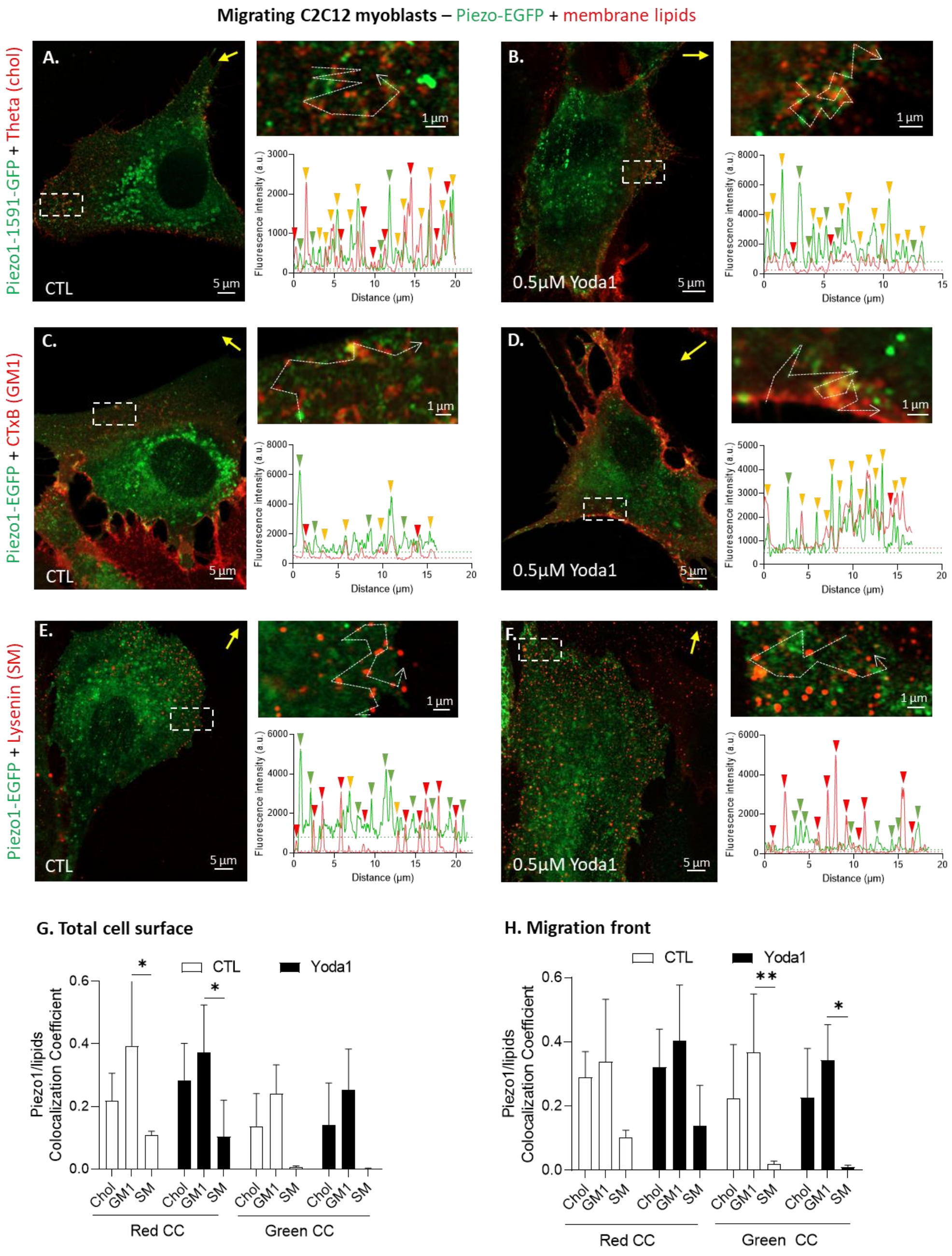

Figure S9. Piezo1 clusters mainly associate with GM1- and chol-enriched domains in migrating myoblasts – Extension of Figure 5. (**A-F**) Cells were transfected with the fluorescent Piezo1-EGFP construct, left untreated (A,C,E) or pretreated with 0.5 µM Yoda1 for 30 minutes (B,D,F), then migrated for 5 hours in Ibidi chambers. Cells were next labeled for chol (Theta, A,B), GM1 (CTxB, C,D) or SM (Lysenin, E,F), visualized by vital confocal microscopy in super-resolution mode, and fluorescence intensity profiles were drawn along the cell periphery to visualize Piezo1 and lipid overlap. Yellow arrows, direction of migration; dotted arrows in insets, profiles; green or red arrowheads, Piezo1 or lipid enrichment; yellow arrowheads, colocalization; dotted lines on graphs, thresholds. (**G,H**) Quantification of the extent of colocalization between Piezo1 and membrane lipids at the total cell surface (G) or at the migration front specifically (H). Data are expressed as the sum of the colocalized pixel count divided by the sum of the colocalized and the red or green pixel counts, which corresponds to the red or green colocalization coefficient (CC), respectively. Two-way ANOVA followed by Sidak’s multiple comparisons test.

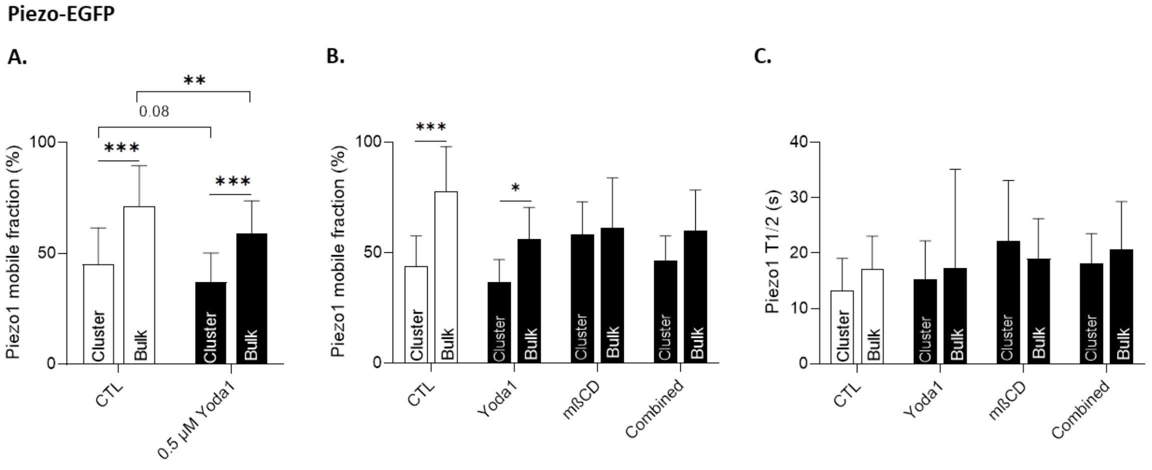

Figure S10. The restriction of membrane lateral diffusion of Piezo1 in clusters is abrogated by cholesterol depletion – Extension of Figures 6 and 8. Cells were transfected with the fluorescent Piezo1-EGFP construct then pretreated for 30 minutes with 0.5 µM Yoda1 (A, n = 32-36 cells from 5 independent experiments), with 5 mM mβCD or a combination of both treatments (B,C, n = 11-15 cells from 3 independent experiments). Photobleaching was done on 2 regions of interest (Piezo1 clusters or bulk membrane) and Piezo1 mobile fraction (A,B) and half-time fluorescence recovery (C) were quantified. Two-way ANOVA followed by Sidak's multiple comparisons test.

Table S1. Sequences of the siRNAs targeting Piezo1.

Name	siRNA ID	Forward	Reverse
**siPiezo1 #1**	AM16708-502463	5'-AGGACAUCGUCACUGCACAtt-3'	5'-UGUGCAGUGACGAUGUCCUca-3'
**siPiezo1 #2**	AM16708-502462	5'-ACUUCGUAGACCUCCCUAAtt-3'	5'-UUAGGGAGGUCUACGAAGUtc-3'

Table S2. Sequences of the designed primers targeting mouse Piezo1 mRNA.

Gene	Forward	Reverse	Size
**Piezo1**	5'-CCTCTTCCTTTTCCAGGGGTTC-3'	5'-GCCTCCCATACCATACTTGACAAT-3'	226bp
